# Association of adolescent self-esteem in 2014 and cognitive performance in 2014, 2016, and 2018: a longitudinal study

**DOI:** 10.3389/fpsyg.2023.1180397

**Published:** 2023-05-02

**Authors:** Xue Wang, Yu Liu, Zhe Zhao, Wenting Liu, Yuqi Chen, Yu Chen, Shuang Zang

**Affiliations:** ^1^Department of Community Nursing, School of Nursing, China Medical University, Shenyang, China; ^2^Institute of International Medical Education, China Medical University, Shenyang, China; ^3^School of Nursing, Southern Medical University, Guangzhou, China

**Keywords:** adolescent, self-esteem, cognitive performance, cognition, longitudinal study

## Abstract

**Background:**

Cognition has long been regarded as a significant factor influencing individuals’ lives. Prior studies have underscored that self-esteem is associated with cognition, and there exists a knowledge gap regarding whether self-esteem remains associated with subsequent cognitive performance during adolescence, a crucial period for neurological development and influencing adult outcomes.

**Methods:**

We conducted this population-based study using longitudinal data stretching three waves (2014, 2016, and 2018) of surveys from the nationally representative China Family Panel Studies (CFPS) to explore the association between adolescents’ self-esteem in 2014 and cognitive performance in 2014, 2016, and 2018.

**Results:**

The results of the present study showed that self-esteem during adolescence in 2014 was significantly associated with cognitive performance in 2014, 2016, and 2018. This association remained robust after an extensive range of covariate adjustments (e.g., adolescents, parental, and family characteristics).

**Conclusion:**

The findings in this study provide further insight into the understanding of the related factors for cognitive development across the life course and highlight the importance of improving individual self-esteem during adolescence.

## Introduction

Adolescent is a crucial period for the development of cognition (Schalbetter et al., 2022). Cognitive performance includes many aspects, such as intelligence, rationality, and memory (Donnelly et al., 2016). Cognitive development is pivotal for adequate self-perception in relation to social environments and for cultivating learning and adaptative skills (Koh, 1982). As an essential determinant of behaviors and mental (Mo et al., 2021), it is very important to measure cognition scientifically. Previous studies have used composite tests in reading, math, science, and history to measure adolescents’ cognitive performance (Marsh and Kleitman, 2003; Ruiz et al., 2010; Travlos, 2010). Following an initial period of cognitive maturation, an individual’s cognitive abilities are not immutable, but rather, subject to variability in response to contextual, developmental, dispositional, temperamental, and other factors (Horn and Cattell, 1966; Cattell, 1974). The implicit theories of intelligence have posited that individuals’ intelligence or ability can be enhanced by the adoption of certain beliefs (e.g., motivation, self-esteem) which may lead to further optimization of personal intelligence (Dweck, 2000).

Good cognitive performance in youth is associated with better school performance, increased employment prospects, and improved quality of life (Ramsden et al., 2011; Vera and Cortés, 2021). In contrast, poor cognitive performance is related to reduced educational attainment, social impairments, impaired career opportunities, poor health conditions, and low self-esteem (Pruessner et al., 2004; Atique-Ur-Rehman and Neill, 2019; Chen et al., 2021). A study of 2,370 secondary school students in Chilean revealed that lower cognitive performance was associated with decreased levels of self-esteem (Huepe et al., 2011). Thus, good cognition is very important for the personal development of adolescents.

Self-esteem is characterized as the comprehensive assessment of an individual’s cognition and affects in relation to the self as an object (Rosenberg, 1965). According to the two-factor model of self-esteem, global self-esteem comprises two components: self-competence and self-liking (Dutton and Cook, 2001). These dimensions involve the evaluation of one’s capabilities and are associated with the process of introspection and the emotional responses to social feedback (Tafarodi and Milne, 2002). The self-consistency theory posits that increased self-esteem facilitates the enhancement of self-perceptions and behaviors (Korman, 1970). Low self-esteem in children and youth is generally associated with negative attitudes and a diminished capacity for coping with daily and developmental challenges (Greenberg et al., 1992). Therefore, children with low self-esteem tend to engage in negative behaviors (e.g., school absences, drinking, and smoking) and are more likely to suffer from psychological disorders such as anxiety and depression (McGee et al., 2001; Donnellan et al., 2005). Additionally, several studies also revealed that low self-esteem may negatively impact adolescents’ social relationships, health, school performance, and cognitive development (Falkner et al., 2001; Harris and Orth, 2020; Zapata-Lamana et al., 2021).

Given that low self-esteem has multiple negative effects on individuals, understanding how the association of adolescent self-esteem with cognitive performance is crucial. Adolescence is an important period for the development of self-esteem and cognitive performance. It is a critical period for individual cognitive, emotional, and social functional development, as well as for the development of self-esteem. Therefore, exploring the association between adolescent self-esteem and cognitive performance can provide important theoretical and practical value for adolescent mental health and academic growth.

The association between self-esteem and cognitive performance among children and adolescents has been confirmed by a body of studies (Martins et al., 2002; Sigfusdottir et al., 2007; Tobia et al., 2017; Zheng et al., 2020). However, while prior research has indicated that self-esteem is a relatively stable construct that is generally formed during childhood or adolescence and has long-lasting effects on subsequent outcomes (Wagner et al., 2016; Saini et al., 2021), it is unclear how this association manifests over time. The theory of self-esteem development in children and adolescents suggests that adolescence is a critical period for self-esteem development, and that the self-esteem during this period can predict subsequent personal development outcomes to a certain extent (James, 1890). Additionally, it has been suggested that self-esteem is related to underlying neural systems, such as neural and computational processes underlying dynamic changes in self-esteem (Will et al., 2017), which then contribute to cognition (Tsvetanov et al., 2016). Moreover, adolescents with high self-esteem tend to engage in cognitive stimulating activities (Zhao et al., 2021), which can enhance their cognitive performance (Wright et al., 2016). To gain a deeper understanding of the association between self-esteem and cognitive performance during adolescence, longitudinal studies are needed. Longitudinal studies can provide valuable insights into the directionality and stability of this association. This can help identify critical periods during which interventions aimed at improving self-esteem and cognitive performance may have the greatest impact.

To address this gap in the literature, the current study aims to investigate the association between self-esteem in 2014 and cognitive performance in 2014, 2016, and 2018 among adolescents aged 10–15 years old using nationally representative data in China. Specifically, we hypothesize that higher self-esteem in 2014 will be associated with better cognitive performance in subsequent years, and that this association will be stable over time.

## Materials and methods

### Study population and data sources

**FIGURE 1:**
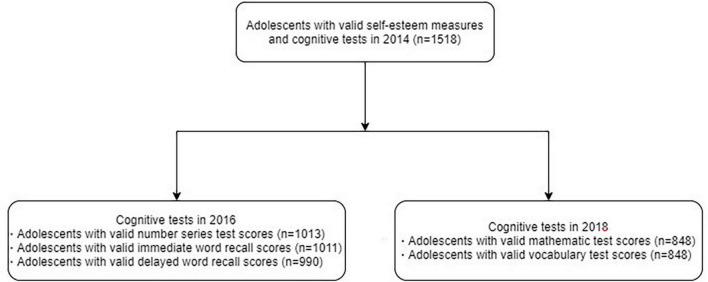
Flow chart of the study population.

The data of this study came from the China Family Panel Studies (CFPS),^1^ a social survey conducted by Peking University’s Institute of Social Science Survey. This survey aims to conduct a longitudinal study of Chinese households, communities, and individuals. The follow-up survey was conducted every two years for CFPS respondents. Approximately 80.64% of the Chinese population was represented in the survey sample, which was drawn from 25 out of 31 provinces/municipalities/autonomous regions. The survey was conducted with a multistage proportional probability sample, involving the sampling of districts/counties, villages/communities, and households. The survey adopted a face-to-face questionnaire and computer-assisted personal interviewing as a tool to improve operational efficiency. This survey employed a suite of quality assurance measures, including telephone verification, field verification, audio recording checks, interview reviews, and statistical analysis to ensure data reliability (Xie and Lu, 2015). The 2014 survey was used as the baseline in our study, and 1518 adolescents aged 10–15 years old made up the sample of the 2014 survey. The participants identified in 2014 are used to track 2016 and 2018, respectively (Figure 1 and Table 1).

**TABLE 1:** The demographic characteristics of the study population by the year.

Variables	2014 (*n* = 1,518)	2016 (*n* = 1,013)	2018 (*n* = 848)
**Adolescents’ characteristics**			
Age in 2014, median (IQR)	11.00 (10.00,15.00)	11.00 (10.00,15.00)	11.00 (10.00,13.00)
Gender (Female), no. (%)	815 (53.69)	535 (52.81)	436 (51.42)
**Parents’ characteristics**			
Maternal age in 2014, median (IQR)	38.00 (35.00,42.00)	–	–
Paternal age in 2014, median (IQR)	40.00 (37.00,44.00)	41.00 (37.00,44.00)	40.00 (37.00,44.00)
Maternal education level in 2014, median (IQR)	7.00 (4.00,9.00)	6.00 (4.00,9.00)	8.00 (4.00,9.00)
Paternal education level in 2014, median (IQR)	9.00 (6.00,9.00)	–	–
Mother was employed in 2014, no. (%)	1,070 (85.88)	753 (86.25)	639 (87.53)
Father was employed in 2014, no. (%)	1,157 (95.86)	776 (96.64)	640 (95.52)
**Household characteristics**			
Lived in urban area in 2014, no. (%)	611 (40.79)	404 (40.36)	347 (41.56)
Household per capita income in 2014 (CNY), median (IQR)	8,012.50 (3,907.14, 13,760.00)	7,944.45 (3,747.50, 13,177.50)	8,159.0 (3,987.500, 13,937.50)
Family size in 2014, median (IQR)	5.00 (4.00, 6.00)	–	–

The CFPS was approved by Peking University’s Biomedical Ethics Review Committee, and all participants provided written informed consent. The ethical approval number was IRB00001052-14010. Permission for the use of data was obtained from the CFPS.

### Procedure of assessment

The socio-economic data of the respondents and their families come from the face-to-face questionnaire survey of the respondents. CFPS survey is not paid to respondents. Cognitive tests have no time limit.

### Exposure measure

#### Self-esteem

The CFPS uses a revised and updated 14-item version of the Rosenberg Self-Esteem Scale (RSES) to measure the individual overall perception of self-esteem (Rosenberg, 1965). The original self-esteem scale consists of 10 questions, of which five questions are positive scoring questions and five questions are negative scoring questions. CFPS adapted the original self-esteem scale into 14 questions, adding additional items: “I can’t solve difficulties,” “I feel compelled by life,” “I have control over things,” and “I am often helpless in life.” RSES has been widely used with good reliability in China and is tailored to the survey of the general population (Tian et al., 2015; Yang et al., 2019). On a five-point Likert scale, respondents selected their level of agreement with the positively rated question (1 = strongly disagree, 2 = disagree, 3 = neither agree nor disagree, 4 = agree, 5 = strongly agree), and negative items were reverse scored. Generally, the higher the total score, the greater the level of self-esteem. The Cronbach’s alpha for this measure was 0.717 in this study.

#### Outcome measures

The CFPS developed two sets of tests to assess cognitive performance in a nationally representative sample of individuals aged 10 years or older (Xie and Hu, 2014). Two sets of cognitive tests include word recall ability (immediate and delayed word recall tests), number sequence ability (number series test) in the 2016 wave of CFPS; word memorization (vocabulary test), and mathematical calculation abilities (mathematics test) in 2014 and 2018 waves of CFPS. The usage of both of these tests has been widely adopted and validated for measuring cognitive ability in Chinese adults and adolescents in previous studies (Zhang et al., 2018; Ren et al., 2019; Li et al., 2021). As for the research process in 2016 CFPS databases, the respondents were shown a randomly presented list of 10 concrete nouns (e.g., rice, river, doctor) and then asked to recall as many of those words as they could in any order. Following approximately 5 min of delay, respondents were asked to recall as many of the original words related to subjective wellbeing as possible after answering 31 questions. For both the immediate and delayed word recall tests, the final test scores were calculated as the number of words correctly recalled by the respondents, ranging from 0 to 10. The number series test is a two-stage adaptive test consisting of three items per stage. Each test item consists of a series of numerical values with one value omitted, and respondents are asked to provide the missing value which accurately follows the numerical pattern. In the initial stage of the survey, all respondents were presented with three items of varying difficulty levels: an easier one, a moderately difficult one, and a more challenging one. Based on the number of correctly answered items in the first stage, respondents were then presented with one of four sets of three items, with varying levels of difficulty. The number series test has a score range of 0–15.

As for the research process in the 2014 and 2018 CFPS, there are 34 word-recognition questions and 24 standardized mathematics questions. All of these questions are drawn from standard textbooks and are ranked according to their difficulty level. Respondents’ ability to answer the initial question depends on their education level, and the test ends after three incorrect answers. A respondent’s final score is the rank of the hardest question he or she correctly answered. The respondents were unaware of the rules before taking the test, thus they would not have deliberately failed it. The mathematics test had a score range of 0–24, the vocabulary test had a score range of 0–34. High scores indicate better performance.

In this study, the word recall ability (immediate and delayed word recall tests) and word memorization (vocabulary test) tests were administered to assess school-related skills including word knowledge and verbal concept formation. While the number sequence ability (number series test) and mathematical calculation abilities (mathematics test) tests were conducted to evaluate the ability to obtain, process, and retain mathematical information.

#### Covariates

We included covariates at the adolescents, parents, and household levels measured in 2014 according to previous research (Wang and Veugelers, 2008; Zhou et al., 2019). The characteristics of adolescents included age (continuous variable) and gender (female vs. male), and the characteristics of the parents included maternal and paternal ages (continuous variables), education levels (years of education, continuous variable), and employment status (have a job vs. have no job). Among the characteristics of the households were urban–rural location (urban vs. rural), household per capita income (continuous variable), and family size (continuous variable).

### Statistical analysis

First, Kolmogorov–Smirnov tests were used to test the distribution of the continuous variables. All the continuous variables were found to be non-normally distributed. Therefore, each continuous variable was reported as a median with a range of interquartile range (IQR). Numbers and percentages (%) were used to express categorical variables.

Second, an analysis of the association between self-esteem and cognitive performance was conducted using the generalized linear model. Unadjusted and adjusted models were developed. In the adjusted model, covariates related to adolescents’ characteristics (age and gender), parents’ characteristics (maternal and paternal ages, maternal and paternal education levels, maternal and paternal employment status), and household characteristics (urban–rural location, household per capita income, and family size) were included. Moreover, variance inflation factors were calculated for each variable to determine whether the generalized linear models were collinear.

Third, we used a smoothing plot to depict the association between self-esteem and cognitive performance, adjusting for potential confounders. Using corresponding modules, threshold effects of confounding factors were also assessed.

In addition, we conducted a series of sensitivity analyses to ensure that the results were robust. First, we calculated the *p* for interaction by stratifying covariates among different subgroups and observing the trend of regression coefficients. We further examined whether the existing missing variables led to bias in our findings by conducting a multiple-imputation analysis. Finally, we calculated *E*-values to examine the possibility of unmeasured confounders between self-esteem and cognitive performance (Haneuse et al., 2019).

*p* < 0.05 was used as the standard of statistical significance, and statistical tests were two-sided. Stata version 16.0 (StataCorp, College Station, TX, USA) was used for the main statistical analyses, while R version 3.6.0 (R Foundation for Statistical Computing, Vienna, Austria) was used in the creation of corresponding figures.

## Results

### Demographic characteristics

We measured the association of adolescents’ self-esteem in 2014 with cognitive performance in 2014, 2016, and 2018, respectively. In 2016, 848 of the 1,518 adolescents identified in the 2014 survey (55.66%) were followed up, and 1,013 (66.73%) of the 1,518 adolescents identified in the 2014 survey were followed up in 2018. In the survey of 2016 and 2018, data attrition was observed as a result of the following reasons, such as participant withdrawal, loss to follow-up, incomplete cognitive assessments, and instances where participants completed the survey but provided responses of “unknown” or “inapplicable” for the cognitive assessments. See Figure 1 and Table 1 for details.

### Association of self-esteem and cognitive performance

The unadjusted generalized linear models showed significant associations between self-esteem and cognitive performance (all *p* < 0.05). Except for the number series test in 2016 (β = 0.04, 95% CI −0.01 to 0.10, *p* = 0.127), other outcomes were still statistically significant after covariate adjustment (all *p* < 0.05). See Table 2 for details. All the variance inflation coefficients of the variables in these models fell below the value of 1.2. Multicollinearity among variables did not appear to be significant.

**TABLE 2:** The association of self-esteem in 2014 with cognitive test scores in 2014, 2016, and 2018.

Items	Mathematics test				Vocabulary test			
	2014 (*n* = 1,518)		2018 (*n* = 848)		2014 (*n* = 1,518)		2018 (*n* = 848)	
	β (95% CI)	*p*	β (95% CI)	*p*	β (95% CI)	*p*	β (95% CI)	*p*
**(a) Mathematics and vocabulary test scores in 2014 and 2018**								
**Unadjusted model**								
Self-esteem in 2014	0.18 (0.14,0.22)	<0.001	0.18 (0.12,0.23)	<0.001	0.36 (0.30,0.43)	<0.001	0.23 (0.17,0.28)	<0.001
**Adjusted model**								
Self-esteem in 2014	0.12 (0.07,0.16)	<0.001	0.09 (0.03,0.15)	0.005	0.22 (0.14,0.30)	<0.001	0.15 (0.08,0.21)	<0.001
Items	Number series test (*n* = 1,013)			Immediate word recall (*n* = 1,011)			Delayed word recall (*n* = 990)	
	**β (95% CI)**		***p***		**β (95% CI)**	***p***	**β (95% CI)**	***p***
**(b) Number series test, immediate word recall, and delayed word recall scores in 2016**								
**Unadjusted model**								
Self-esteem in 2014	0.09 (0.05,0.13)		<0.001		0.04 (0.02,0.06)	<0.001	0.04 (0.01,0.06)	0.002
**Adjusted model**								
Self-esteem in 2014	0.04 (−0.01,0.10)		0.127		0.03 (0,0.05)	0.030	0.05 (0.02,0.08)	0.002

In the adjusted model, this table controlled for adolescents’ characteristics (age and gender), parents’ characteristics (maternal age, paternal age, maternal education level, paternal education level, maternal employment status, paternal employment status), and household characteristics (urban–rural location, household per capita income, family size).

**FIGURE 2:**
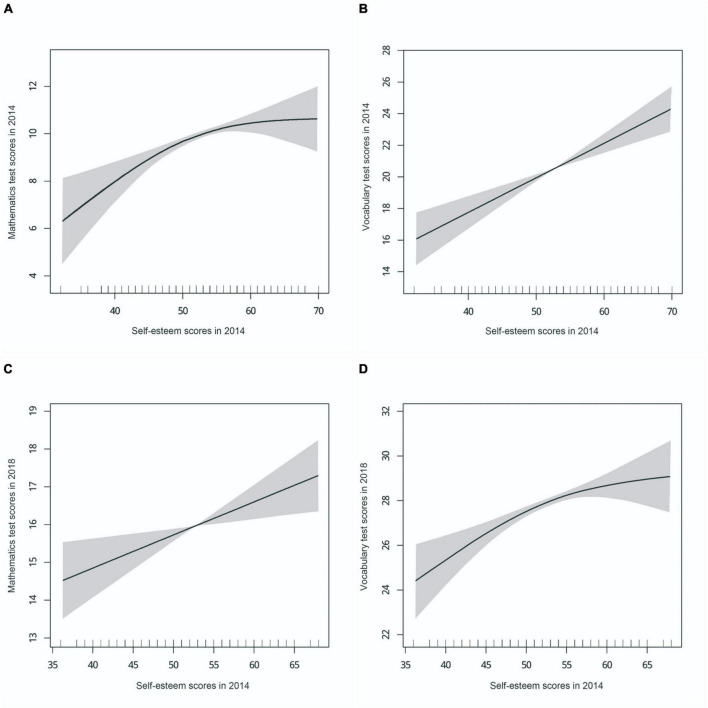
The association of self-esteem in 2014 with cognitive test scores in 2014 and 2018. **(A)** Association of self-esteem scores in 2014 with mathematics test scores in 2014. **(B)** Association of self-esteem scores in 2014 with vocabulary test scores in 2014. **(C)** Association of self-esteem scores in 2014 with mathematics test scores in 2018. **(D)** Association of self-esteem scores in 2014 with vocabulary test scores in 2018. Adjusted for adolescents’ characteristics (age and gender), parents’ characteristics (maternal age, paternal age, maternal education level, paternal education level, maternal employment status, paternal employment status), and household characteristics (urban–rural location, household per capita income, family size). Solid lines represent the spline curve, and the shaded areas represent the 95% confidence interval of the spline curve.

**FIGURE 3:**
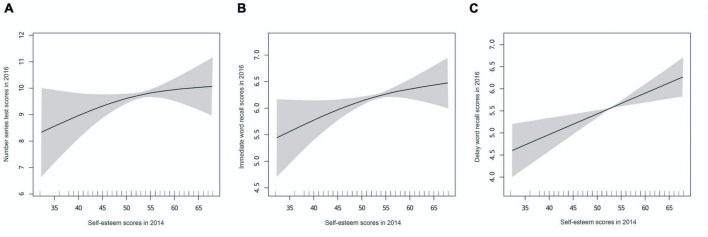
The association of self-esteem in 2014 with cognitive test scores in 2016. **(A)** Association of self-esteem scores in 2014 with number series test scores in 2016. **(B)** Association of self-esteem scores in 2014 with immediate word recall scores in 2016. **(C)** Association of self-esteem scores in 2014 with delay word recall scores in 2016. Adjusted for adolescents’ characteristics (age and gender), parents’ characteristics (maternal age, paternal age, maternal education level, paternal education level, maternal employment status, paternal employment status), and household characteristics (urban–rural location, household per capita income, family size). Solid lines represent the spline curve, and the shaded areas represent the 95% confidence interval of the spline curve.

After adjusting for potential confounders, there was a positive linear association between self-esteem and the following outcomes: vocabulary test in 2014, immediate word recall in 2016, delayed word recall in 2016, and mathematics test in 2018 (all *p* < 0.05 by using linear regression model). See Figures 2, 3, and Supplementary Table 1 for details. The final model exhibited a range of values for the Akaike Information Criterion (AIC) between 3.74 and 6.60, and for the Bayesian Information Criterion (BIC) between −2,615.62 and 32,848.14. As demonstrated by the two-piece piecewise regression models of the non-linear association between the self-esteem and mathematics test in 2014 and the vocabulary test in 2018, the estimated highest point was 45 for both (*p* for likelihood ratio test < 0.05). In the threshold effect analysis, among the population with self-esteem scores < 45, the mathematics test scores increased significantly in 2014 (β = 0.48, 95% CI 0.24–0.71, *p* < 0.001). Among the population with self-esteem scores ≥ 45, the mathematics test scores in 2014 showed a significant increase in moderation (β = 0.06, 95% CI 0–0.11, *p* = 0.045). Additionally, the piecewise regression model observed that self-esteem scores and number series test scores in 2016 had an approximately curvilinear relationship (*p* for likelihood ratio test = 0.056).

### Sensitivity analyses

In the first sensitivity analysis, stratified analysis was carried out to examine the association between self-esteem and cognitive performance in different subgroups (adolescents’ age, adolescents’ gender, maternal age, paternal age, maternal education level, paternal education level, maternal employment status, paternal employment status, urban–rural location, and household per capita income). In most subgroups, the stratified analysis showed a correlation between self-esteem and outcomes. For example, for adolescents aged 10–12 years old (β = 0.13, 95% CI 0.10–0.17, *p* < 0.001) or aged 13–15 years old (β = 0.20, 95% CI 0.12–0.27, *p* < 0.001) in 2014, female (β = 0.15, 95% CI 0.10–0.20, *p* < 0.001) and male (β = 0.21, 95% CI 0.14–0.27, *p* < 0.001), self-esteem was related to mathematics test scores in 2014. However, the correlation between self-esteem and outcome was not statistically significant in several subgroups. For example, adolescents whose paternal age was 46 years or older (β = 0.06, 95% CI −0.09to 0.21, *p* = 0.413), maternal age was 46 years or older (β = 0.17, 95% CI −0.04 to 0.38, *p* = 0.117). The correlation between self-esteem and mathematics test scores in 2018 was not statistically significant. According to the interaction test, the self-esteem effect on cognitive performance was not significantly affected by most variables in 2014, 2016, and 2018 (*p* for interaction > 0.05). However, there are several exceptions. For example, the impact of self-esteem on the vocabulary test in 2018 is influenced by factors such as paternal age (*p* for interaction = 0.027), maternal education level (*p* for interaction = 0.011), parental employment status (*p* for interaction = 0.041), and urban–rural location (*p* for interaction = 0.038). See Supplementary Table 2 for details.

The second sensitivity analysis was performed by imputed covariates and test scores for people with invalid data or those who were lost to follow-up. Most of the outcomes remained robust (*p* < 0.05). The only exception was that after adjusting for confounding variables, the association between self-esteem and the number series test in 2016 became significant (β = 0.06, 95% CI 0.02–0.11, *p* = 0.004). See Supplementary Table 3 for details.

For the final sensitivity analysis, we calculated *E*-values as a measure of the sensitivity to confounding factors that were not measured. The *E*-values for the mathematics and vocabulary tests were 4.29 and 4.27 in 2014, respectively. The *E*-values for the mathematics and vocabulary tests in 2018 were 4.31 and 4.26. The number series test, immediate word recall, and delayed word recall of *E*-values in 2016 were 4.36, 4.32, and 4.29, respectively. The above higher *E*-values demonstrate a less likely possibility that unobserved confounders can explain the observed association between self-esteem and cognition outcomes.

## Discussion

To our best knowledge, this study is the first systematic examination of the association between adolescents’ self-esteem in 2014 and cognitive performance in 2014, 2016, and 2018 by using developmental data. We found that self-esteem in adolescents 10–15 years old was positively associated with cognitive performance until four years later.

Our study found that self-esteem was associated with cognitive performance. Specifically, adolescents’ self-esteem in 2014 was positively associated with cognitive performance in 2014, 2016, and 2018. Although there is a lack of previous research on adolescents’ self-esteem and cognitive performance. Much evidence on adolescents’ self-esteem and academic performance exists. It is worth noting that the constituent indicators of academic performance in previous studies are also part of the cognitive performance of this study. For instance, a study from Bowles showed that there was an association between self-esteem and students’ math test in the last semester (Bowles, 1999). Kugle et al. (1983) found that there was a strong association between the scores on the vocabulary reading tests and the level of self-esteem in children. According to the findings of Zapata-Lamana et al. (2021), adolescents with lower levels of self-esteem tend to perceive their cognitive abilities as subpar, as evidenced by their self-reported deficits in memory retention, slower problem-solving skills in mathematical contexts, increased difficulties in executing complex tasks, and greater challenges in maintaining focus during classroom activities. Variations in learning and academic achievement levels among adolescents in the school stage can be attributed to differences in cognitive abilities (Martins et al., 2016). Research suggested that cognitive abilities such as working memory, attention, and processing speed are crucial in facilitating learning and academic achievement (Moxley-Paquette and Burkholder, 2020; Esposito and Bauer, 2022). For instance, individuals with higher working memory capacity can retain and manipulate more information in their minds, which facilitates the learning process (Allen et al., 2021; Cotton and Ricker, 2022). Similarly, individuals with better attentional control can sustain their focus on the task at hand for a more extended period, leading to improved learning outcomes (May et al., 2013; Isbell et al., 2018). Moreover, cognitive abilities are also known to impact other factors that influence academic achievement, such as motivation and self-regulation. For example, individuals with better self-regulation skills can manage their time more effectively, avoid distractions, and persist with tasks until completion, which can lead to better academic performance (Sandars and Cleary, 2011; Sahranavard et al., 2018). Although there is a lack of direct research on the relationship between self-esteem and cognitive performance, there is evidence to suggest an indirect association between self-esteem and cognitive performance. Specifically, the association between self-esteem and academic achievement, as well as the strong association between cognitive ability and academic performance, provide evidence to support a potential link between self-esteem and cognitive performance. Thus, the association between adolescents’ self-esteem and cognitive performance in our study emphasized the need for greater attention to be given to the role of self-esteem in the development and maintenance of cognitive abilities, particularly during adolescence when both self-esteem and cognitive functioning undergo significant changes.

In this study, curve-fitting plots revealed a positive association between adolescent self-esteem in 2014 and cognitive performance in 2014, 2016, and 2018. There is little clear evidence as to how adolescents’ self-esteem was associated with their cognitive performance, but the underlying related theories may help to explain this association. The self-fulfilling prophecy theory suggests that individuals’ beliefs regarding themselves can have a powerful influence on their behavior and performance (Carvalho et al., 2018). According to this theory, individuals possessing high self-esteem may be more inclined to engage in activities that foster cognitive development (Madon et al., 1997), thus resulting in superior cognitive performance over time. Conversely, individuals possessing low self-esteem may be more likely to engage in activities that hinder cognitive development, leading to suboptimal cognitive performance over time. In addition, the social cognitive theory indicates that individuals’ self-beliefs and motivation significantly impact their behavior and performance (Bandura, 2001). Specifically, individuals with high self-esteem tend to have positive self-concepts, leading to a sense of self-efficacy and motivation to engage in challenging activities that promote cognitive development. This, in turn, can lead to superior cognitive performance. Conversely, individuals with low self-esteem may experience negative self-concepts, leading to a lack of self-efficacy and motivation to engage in challenging activities that foster cognitive development, resulting in suboptimal cognitive performance. As well, the concept of cognitive reserve is also relevant to this finding. Cognitive reserve refers to the accumulation of knowledge, experience, skills, and intelligence that an individual acquires throughout their lifespan (Stern, 2002). It encompasses a range of knowledge and abilities acquired through learning and experiences, including vocabulary, learning ability, computational skills, attention, working memory, problem-solving ability, and comprehension (Fyffe et al., 2011; Stern et al., 2020). According to this concept, individuals who engage in activities that promote cognitive development, such as those with high self-esteem, may have a greater cognitive reserve, which can improve cognitive performance over time. These related theories could help to explain our results and provide an additional view to understanding the underlying association between adolescents’ self-esteem and cognitive performance.

The current study has some limitations that need to be mentioned. First, similar to other large-scale longitudinal studies, the sample in this study is affected by attrition that occurred over time. Second, a total of 665 participants passed all 3 waves. We only use 2014 as the baseline to track the cognitive performance of adolescents in 2016 and 2018, respectively. Future research should include only those who participate in all wave assessments in the analysis to evaluate the continuous changes in adolescents’ cognitive performance in subsequent years. Third, we only investigated self-esteem in 2014 but did not investigate self-esteem in subsequent years. Future studies should follow up the self-esteem longitudinally to evaluate the change in self-esteem. Fourth, our study did not adjust for baseline cognitive performance in our longitudinal analysis. Future studies should consider adjusting for baseline cognitive performance in longitudinal analysis. Fifth, the assessment tool for adolescents’ self-esteem is based on their self-report and therefore individuals’ perceptions of self-esteem may be prone to bias. Sixth, the main idea of this study did not include the internal correlation of cognitive outcome measurement items. Future research should consider verifying the internal correlation between cognitive outcomes. Seventh, although the *E*-values of this study indicate that unobserved confounding is less likely to explain observed associations between adolescents’ self-esteem and cognitive performance, there still may be bias resulting from the remaining unobserved confounding (e.g., family socioeconomic status). Eighth, based on prior research (Kang, 2023), it is essential to consider the potential influence of personality traits on the results of this study. As the current study utilizes the CFPS database, which does not measure personality traits, we cannot exclude the possibility of such traits having an impact on our findings. Therefore, future studies investigating the association between self-esteem and cognitive performance in adolescents should take into account the potential influence of personality traits. Finally, we use the commonly used evaluation metrics in China to measure cognitive performance, but cognitive performance is a complex and comprehensive metric, and for example, inhibition control (Kang et al., 2022) has not been evaluated in this study, so a more comprehensive evaluation may be needed.

## Conclusion

The results of our study suggest that adolescent self-esteem in 2014 is associated with cognitive performance in 2014, 2016, and 2018. The findings extend the understanding of the association between adolescent self-esteem and cognitive performance and highlight the importance of fostering positive self-esteem in adolescents, as it may have long-term benefits for cognitive performance. Based on these findings, improving individual self-esteem during adolescence may thus greatly broaden the window of opportunity for cognitive development.

## Data availability statement

Publicly available datasets were analyzed in this study. This data can be found here: Original data in this study are obtained from the Institute of Social Science Survey of Peking University and are available at https://www.isss.pku.edu.cn/cfps/index.htm.

## Ethics statement

The CFPS was approved by the Peking University’s Biomedical Ethics Review Committee, and all participants provided written informed consent. The ethical approval number was IRB00001052-14010.

## Author contributions

XW, SZ, and YC: conceptualization. XW: methodology and writing—original draft preparation. XW, YL, ZZ, WL, YQC, YC, and SZ: writing—review and editing. SZ: data acquisition. All authors read and agreed to the published version of the manuscript.

## Footnotes

1
https://www.isss.pku.edu.cn/cfps/
