# Supplementary material for: Association of adolescent self-esteem in 2014 and cognitive performance in 2014, 2016, and 2018: a longitudinal study
Source: Front Psychol. 2023 May 2;14:1180397. doi: 10.3389/fpsyg.2023.1180397 (PMC10185744; doi:10.3389/fpsyg.2023.1180397)
Supplement: Supplementary file 1 [file Data_Sheet_1.docx]

**Table S1.** **Threshold effect analyses of self-esteem on cognitive test scores in 2014, 2016, and 2018**

**(a) Mathematics and vocabulary test scores in 2014 and 2018**

| **Items** | **Mathematics test** | | | | **Vocabulary test** | | | |
| --- | --- | --- | --- | --- | --- | --- | --- | --- |
|  | **2014 (*n*=1518)** | | **2018 (*n*=848)** | | **2014 (*n*=1518)** | | **2018 (*n*=848)** | |
|  | ***β* (95%CI)** | ***p*** | ***β* (95%CI)** | ***p*** | ***β* (95%CI)** | ***p*** | ***β* (95%CI)** | ***p*** |
| **Linear regression model** | 0.11 (0.06, 0.15) | <0.001 | 0.09 (0.03, 0.15) | 0.006 | 0.22 (0.14, 0.30) | <0.001 | 0.15 (0.08, 0.21) | <0.001 |
| **Nonlinear model, regression coefficients (*β*)** |  |  |  |  |  |  |  |  |
| Break point of self-esteem (*K*) | 45 | | 58 | | 60 | | 45 | |
| <K | 0.48 (0.24, 0.71) | <0.001 | 0.12 (0.04, 0.19) | 0.002 | 0.25 (0.15, 0.34) | <0.001 | 0.56 (0.22, 0.90) | 0.002 |
| **≥**K | 0.06 (0, 0.11) | 0.045 | -0.08 (-0.33, 0.17) | 0.519 | -0.03 (-0.45, 0.39) | 0.884 | 0.10 (0.02, 0.18) | 0.010 |
| Difference of *β*-value between strata | -0.42 (-0.68, -0.16) | 0.002 | -0.20 (-0.47, 0.08) | 0.167 | -0.28 (-0.74, 0.18) | 0.238 | -0.46 (-0.84, -0.08) | 0.018 |
| Predicted value of cognitive test scores at break point | 8.83 (8.23, 9.44) | | 17.19 (16.58, 17.79) | | 23.55 (22.58, 24.52) | | 26.74 (26.03, 27.45) | |
| P value for likelihood ratio test | 0.002 | | 0.161 | | 0.234 | | 0.016 | |

1. **Number series test, immediate word recall, and delayed word recall scores in 2016**

| **Items** | **Number series test (*n*=1013)** | | **Immediate word recall (*n*=1011)** | | **Delayed word recall (*n*=990)** | |
| --- | --- | --- | --- | --- | --- | --- |
|  | ***β* (95%CI)** | ***p*** | ***β* (95%CI)** | ***p*** | ***β* (95%CI)** | ***p*** |
| **Linear regression model** | 0.04 (-0.01, 0.10) | 0.127 | 0.03 (0, 0.05) | 0.031 | 0.05 (0.02, 0.08) | 0.002 |
| **Nonlinear model, regression coefficients (*β*)** |  |  |  |  |  |  |
| Break point of self-esteem (*K*) | 59 | | 54 | | 54 | |
| <K | 0.08 (0.01, 0.14) | 0.022 | 0.05 (0.01, 0.08) | 0.017 | 0.06 (0.02, 0.11) | 0.006 |
| **≥**K | -0.20 (-0.45, 0.06) | 0.129 | 0 (-0.06, 0.05) | 0.897 | 0.02 (-0.04, 0.08) | 0.497 |
| Difference of *β*-value between strata | -0.27 (-0.56, 0.01) | 0.059 | -0.05 (-0.12, 0.03) | 0.196 | -0.04 (-0.13, 0.05) | 0.369 |
| Predicted value of cognitive test scores at break point | 10.41 (9.89, 10.93) | | 6.30 (6.12, 6.49) | | 5.66 (5.44, 5.88) | |
| P value for likelihood ratio test | 0.056 | | 0.191 | | 0.364 | |

Note: Adjusted for adolescents’ characteristics (age and gender), parents’ characteristics (maternal age, paternal age, maternal education level, paternal education level, maternal employment status, paternal employment status), and household characteristics (urban‒rural location, household per capita income, family size).

**Table S2. Stratified analyses for the association between self-esteem and cognitive test scores in 2014, 2016, and 2018**

1. **Mathematics and vocabulary test scores in 2014 and 2018**

| **Items** | **Mathematics test** | | | | | | **Vocabulary test** | | | | | |
| --- | --- | --- | --- | --- | --- | --- | --- | --- | --- | --- | --- | --- |
|  | **2014 (*n*=1518)** | | | **2018 (*n*=848)** | | | **2014 (*n*=1518)** | | | **2018 (*n*=848)** | | |
|  | ***β* (95%CI)** | ***p*** | ***p* for interaction** | ***β* (95%CI)** | ***p*** | ***p* for interaction** | ***β* (95%CI)** | ***p*** | ***p* for interaction** | ***β* (95%CI)** | ***p*** | ***p* for interaction** |
| **Age** |  |  | 0.099 |  |  | 0.769 |  |  | 0.253 |  |  | 0.357 |
| 10-12 | 0.13 (0.10, 0.17) | <0.001 |  | 0.16 (0.10, 0.21) | <0.001 |  | 0.31 (0.24, 0.39) | <0.001 |  | 0.20 (0.15, 0.26) | <0.001 |  |
| 13-15 | 0.20 (0.12, 0.27) | <0.001 |  | 0.17 (0.07, 0.28) | 0.002 |  | 0.39 (0.27, 0.50) | <0.001 |  | 0.26 (0.14, 0.38) | <0.001 |  |
| **Gender** |  |  | 0.175 |  |  | 0.275 |  |  | 0.046 |  |  | 0.286 |
| Female | 0.15 (0.10, 0.20) | <0.001 |  | 0.14 (0.07, 0.22) | <0.001 |  | 0.30 (0.21, 0.39) | <0.001 |  | 0.18 (0.11, 0.25) | <0.001 |  |
| Male | 0.21 (0.14, 0.27) | <0.001 |  | 0.20 (0.13, 0.28) | <0.001 |  | 0.44 (0.34, 0.55) | <0.001 |  | 0.24 (0.16, 0.33) | <0.001 |  |
| **Maternal age** |  |  | 0.622 |  |  | 0.626 |  |  | 0.837 |  |  | 0.357 |
| ≤35 | 0.17 (0.12, 0.23) | <0.001 |  | 0.14 (0.05, 0.22) | 0.002 |  | 0.34 (0.23, 0.45) | <0.001 |  | 0.24 (0.15, 0.32) | <0.001 |  |
| 36-40 | 0.18 (0.10, 0.25) | <0.001 |  | 0.21 (0.10, 0.31) | <0.001 |  | 0.40 (0.27, 0.53) | <0.001 |  | 0.16 (0.09, 0.24) | <0.001 |  |
| 41-45 | 0.22 (0.12, 0.32) | <0.001 |  | 0.23 (0.11, 0.34) | <0.001 |  | 0.32 (0.16, 0.49) | <0.001 |  | 0.29 (0.16, 0.43) | <0.001 |  |
| ≥46 | 0.12 (-0.02, 0.25) | 0.087 |  | 0.17 (-0.04, 0.38) | 0.117 |  | 0.40 (0.17, 0.62) | <0.001 |  | 0.23 (-0.01, 0.47) | 0.061 |  |
| **Paternal age** |  |  | 0.951 |  |  | 0.186 |  |  | 0.124 |  |  | 0.027 |
| ≤35 | 0.16 (0.05, 0.27) | 0.005 |  | 0.14 (0, 0.27) | 0.047 |  | 0.28 (0.09, 0.46) | 0.003 |  | 0.19 (0.05, 0.32) | 0.007 |  |
| 36-40 | 0.19 (0.11, 0.27) | <0.001 |  | 0.25 (0.14, 0.36) | <0.001 |  | 0.41 (0.28, 0.54) | <0.001 |  | 0.23 (0.13, 0.32) | <0.001 |  |
| 41-45 | 0.18 (0.11, 0.26) | <0.001 |  | 0.21 (0.11, 0.32) | <0.001 |  | 0.48 (0.35, 0.61) | <0.001 |  | 0.39 (0.26, 0.51) | <0.001 |  |
| ≥46 | 0.16 (0.06, 0.26) | 0.003 |  | 0.06 (-0.09, 0.21) | 0.413 |  | 0.26 (0.08, 0.44) | 0.005 |  | 0.15 (0.01, 0.29) | 0.036 |  |
| **Maternal education level** |  |  | 0.056 |  |  | 0.208 |  |  | 0.015 |  |  | 0.011 |
| ≤6 years | 0.22 (0.16, 0.28) | <0.001 |  | 0.19 (0.11, 0.28) | <0.001 |  | 0.44 (0.33, 0.55) | <0.001 |  | 0.27 (0.17, 0.36) | <0.001 |  |
| 7-9 years | 0.10 (0.02, 0.18) | 0.011 |  | 0.10 (0.01, 0.18) | 0.035 |  | 0.25 (0.12, 0.37) | <0.001 |  | 0.16 (0.09, 0.24) | <0.001 |  |
| ≥10years | 0.13 (0.01, 0.24) | 0.032 |  | 0.09 (-0.03, 0.20) | 0.145 |  | 0.19 (0.03, 0.35) | 0.023 |  | 0.04 (-0.06, 0.13) | 0.479 |  |
| **Paternal education level** |  |  | 0.307 |  |  | 0.622 |  |  | 0.448 |  |  | 0.054 |
| ≤6 years | 0.19 (0.11, 0.27) | <0.001 |  | 0.22 (0.12, 0.32) | <0.001 |  | 0.44 (0.31, 0.57) | <0.001 |  | 0.35 (0.23, 0.46) | <0.001 |  |
| 7-9 years | 0.20 (0.13, 0.27) | <0.001 |  | 0.17 (0.08, 0.26) | <0.001 |  | 0.33 (0.21, 0.44) | <0.001 |  | 0.18 (0.09, 0.27) | <0.001 |  |
| ≥10years | 0.11 (0.01, 0.21) | 0.028 |  | 0.14 (0.01, 0.27) | 0.033 |  | 0.38 (0.22, 0.54) | <0.001 |  | 0.23 (0.13, 0.34) | <0.001 |  |
| **Mother was employed** |  |  | 0.940 |  |  | 0.288 |  |  | 0.343 |  |  | 0.986 |
| Yes | 0.18 (0.13, 0.23) | <0.001 |  | 0.18 (0.11, 0.24) | <0.001 |  | 0.36 (0.28, 0.44) | <0.001 |  | 0.17 (0.11, 0.23) | <0.001 |  |
| No | 0.18 (0.06, 0.31) | 0.005 |  | 0.08 (-0.07, 0.24) | 0.308 |  | 0.25 (0.06, 0.45) | 0.014 |  | 0.17 (0.03, 0.31) | 0.017 |  |
| **Father was employed** |  |  | 0.675 |  |  | 0.780 |  |  | 0.602 |  |  | 0.041 |
| Yes | 0.17 (0.12, 0.21) | <0.001 |  | 0.19 (0.12, 0.25) | <0.001 |  | 0.36 (0.28, 0.43) | <0.001 |  | 0.24 (0.18, 0.30) | <0.001 |  |
| No | 0.22 (0, 0.44) | 0.057 |  | 0.14 (-0.16, 0.43) | 0.363 |  | 0.46 (0.15, 0.77) | 0.006 |  | 0.57 (0.18, 0.96) | 0.008 |  |
| **Urban‒rural** **location** |  |  | 0.035 |  |  | 0.169 |  |  | 0.223 |  |  | 0.038 |
| Rural | 0.21 (0.16, 0.26) | <0.001 |  | 0.13 (0.04, 0.22) | 0.006 |  | 0.40 (0.32, 0.49) | <0.001 |  | 0.28 (0.21, 0.35) | <0.001 |  |
| Urban | 0.12 (0.05, 0.19) | <0.001 |  | 0.21 (0.14, 0.28) | <0.001 |  | 0.32 (0.20, 0.43) | <0.001 |  | 0.16 (0.07, 0.24) | <0.001 |  |
| **Household per capita income** |  |  | 0.226 |  |  | 0.419 |  |  | 0.056 |  |  | 0.204 |
| ≤15981 | 0.19 (0.15, 0.24) | <0.001 |  | 0.19 (0.12, 0.25) | <0.001 |  | 0.40 (0.32, 0.48) | <0.001 |  | 0.25 (0.18, 0.32) | <0.001 |  |
| ＞15981 | 0.12 (0.02, 0.23) | 0.020 |  | 0.13 (0.01, 0.24) | 0.029 |  | 0.22 (0.06, 0.38) | 0.007 |  | 0.16 (0.06, 0.26) | 0.003 |  |

1. **Number series test, immediate word recall, and delayed word recall scores in 2016**

| **Items** | **Number series test (*n*=1013)** | | | **Immediate word recall (*n*=1011)** | | | **Delayed word recall (*n*=990)** | | |
| --- | --- | --- | --- | --- | --- | --- | --- | --- | --- |
|  | ***β* (95%CI)** | ***p*** | ***p* for interaction** | ***β* (95%CI)** | ***p*** | ***p* for interaction** | ***β* (95%CI)** | ***p*** | ***p* for interaction** |
| **Age** |  |  | 0.105 |  |  | 0.537 |  |  | 0.868 |
| 10-12 | 0.11 (0.06, 0.16) | <0.001 |  | 0.04 (0.02, 0.06) | <0.001 |  | 0.04 (0.01, 0.06) | 0.006 |  |
| 13-15 | 0.03 (-0.06, 0.12) | 0.478 |  | 0.03 (-0.01, 0.06) | 0.118 |  | 0.04 (0, 0.08) | 0.063 |  |
| **Gender** |  |  | 0.029 |  |  | 0.646 |  |  | 0.962 |
| Female | 0.05 (-0.01, 0.11) | 0.078 |  | 0.03 (0.01, 0.06) | 0.010 |  | 0.04 (0.01, 0.06) | 0.021 |  |
| Male | 0.15 (0.08, 0.21) | <0.001 |  | 0.04 (0.01, 0.07) | 0.005 |  | 0.03 0, 0.07) | 0.054 |  |
| **Maternal age** |  |  | 0.262 |  |  | 0.722 |  |  | 0.039 |
| ≤35 | 0.06 (-0.02, 0.14) | 0.119 |  | 0.03 (0, 0.07) | 0.054 |  | 0.02 (-0.02, 0.06) | 0.261 |  |
| 36-40 | 0.14 (0.06, 0.23) | <0.001 |  | 0.03 (-0.01, 0.06) | 0.102 |  | 0.04 (-0.01, 0.08) | 0.089 |  |
| 41-45 | 0.08 (-0.01, 0.17) | 0.068 |  | 0.05 (0.01, 0.09) | 0.009 |  | 0.08 (0.04, 0.13) | <0.001 |  |
| ≥46 | -0.01 (-0.16, 0.14) | 0.905 |  | 0.01 (-0.06, 0.08) | 0.776 |  | -0.05 (-0.15, 0.04) | 0.297 |  |
| **Paternal age** |  |  | 0.278 |  |  | 0.809 |  |  | 0.797 |
| ≤35 | 0.16 (0.05, 0.27) | 0.004 |  | 0.02 (-0.03, 0.07) | 0.495 |  | 0.05 (0, 0.11) | 0.070 |  |
| 36-40 | 0.13 (0.04, 0.22) | 0.005 |  | 0.05 (0.01, 0.082) | 0.010 |  | 0.05 (0.01, 0.09) | 0.031 |  |
| 41-45 | 0.05 (-0.03, 0.14) | 0.215 |  | 0.03 (-0.01, 0.07) | 0.012 |  | 0.02 (-0.03, 0.07) | 0.382 |  |
| ≥46 | 0.04 (-0.06, 0.14) | 0.430 |  | 0.04 (-0.01, 0.08) | 0.081 |  | 0.03 (-0.02, 0.08) | 0.246 |  |
| **Maternal education level** |  |  | 0.830 |  |  | 0.820 |  |  | 0.923 |
| ≤6 years | 0.06 (-0.01, 0.12) | 0.081 |  | 0.03 (0.01, 0.06) | 0.024 |  | 0.03 (0, 0.07) | 0.064 |  |
| 7-9 years | 0.08 (0, 0.15) | 0.054 |  | 0.03 (0, 0.06) | 0.075 |  | 0.02 (-0.02, 0.07) | 0.265 |  |
| ≥10years | 0.10 (-0.01, 0.20) | 0.075 |  | 0.02 (-0.02, 0.06) | 0.419 |  | 0.04 (-0.02, 0.09) | 0.175 |  |
| **Paternal education level** |  |  | 0.855 |  |  | 0.682 |  |  | 0.770 |
| ≤6 years | 0.07 (-0.01, 0.15) | 0.075 |  | 0.02 (-0.01, 0.06) | 0.182 |  | 0.03 (-0.02, 0.07) | 0.230 |  |
| 7-9 years | 0.09 (0.02, 0.16) | 0.018 |  | 0.04 (0.01, 0.07) | 0.007 |  | 0.05 (0.01, 0.08) | 0.012 |  |
| ≥10years | 0.11 (0, 0.21) | 0.054 |  | 0.04 (0, 0.09) | 0.070 |  | 0.03 (-0.03, 0.09) | 0.280 |  |
| **Mother was employed** |  |  | 0.880 |  |  | 0.598 |  |  | 0.649 |
| Yes | 0.07 (0.02, 0.12) | 0.004 |  | 0.03 (0.01, 0.05) | 0.007 |  | 0.03 (0, 0.06) | 0.025 |  |
| No | 0.08 (-0.03, 0.20) | 0.160 |  | 0.05 (-0.01, 0.10) | 0.093 |  | 0.05 (-0.01, 0.11) | 0.184 |  |
| **Father was employed** |  |  | 0.573 |  |  | 0.782 |  |  | 0.550 |
| Yes | 0.10 (0.05, 0.15) | <0.001 |  | 0.03 (0.01, 0.06) | 0.002 |  | 0.04 (0.01, 0.07) | 0.003 |  |
| No | 0.01 (-0.30, 0.32) | 0.955 |  | 0.02 (-0.15, 0.18) | 0.865 |  | 0.09 (-0.09, 0.27) | 0.334 |  |
| **Urban‒rural** **location** |  |  | 0.496 |  |  | 0.545 |  |  | 0.355 |
| Rural | 0.08 (0.02, 0.13) | 0.005 |  | 0.03 (0.01, 0.06) | 0.006 |  | 0.03 (0, 0.06) | 0.053 |  |
| Urban | 0.11 (0.04, 0.18) | 0.004 |  | 0.05 (0.01, 0.08) | 0.005 |  | 0.05 (0.01, 0.09) | 0.010 |  |
| **Household per capita income** |  |  | 0.646 |  |  | 0.518 |  |  | 0.508 |
| ≤15981 | 0.08 (0.03, 0.13) | 0.001 |  | 0.04 (0.02, 0.06) | <0.001 |  | 0.04 (0.01, 0.06) | 0.003 |  |
| ＞15981 | 0.11 (0, 0.22) | 0.057 |  | 0.06 (0.01, 0.10) | 0.016 |  | 0.06 (0.01, 0.11) | 0.030 |  |

**Note:** According to China’s National Bureau of Statistics, the national median per capita disposable income was 15981 CNY in 2014. Therefore, we used 15981 CNY as the cutoff point for household per capita income in the stratified analysis. The average annual exchange rate in 2014 was 1 Chinese Yuan = 0.144 US dollars.

**Table S3.** **Using multiple imputations to impute missing values for variables in 2014, 2016, and 2018**

**(a) Mathematics and vocabulary test scores in 2014 and 2018**

| **Items** | **Mathematics test** | | | | **Vocabulary test** | | | |
| --- | --- | --- | --- | --- | --- | --- | --- | --- |
|  | **2014 (*n*=1518)** | | **2018 (*n*=848)** | | **2014 (*n*=1518)** | | **2018 (*n*=848)** | |
|  | ***β* (95%CI)** | ***p*** | ***β* (95%CI)** | ***p*** | ***β* (95%CI)** | ***p*** | ***β* (95%CI)** | ***p*** |
| **Unadjusted model** |  |  |  |  |  |  |  |  |
| Self-esteem in 2014 | 0.18 (0.14,0.22) | <0.001 | 0.18 (0.12,0.23) | <0.001 | 0.37 (0.30,0.44) | <0.001 | 0.23 (0.17,0.28) | <0.001 |
| **Adjusted model** |  |  |  |  |  |  |  |  |
| Self-esteem in 2014 | 0.12 (0.08,0.15) | <0.001 | 0.09 (0.04,0.14) | <0.001 | 0.26 (0.20,0.32) | <0.001 | 0.17 (0.12,0.23) | <0.001 |

**(b) Number series test, immediate word recall, and delayed word recall scores in 2016**

| **Items** | **Number series test (*n*=1013)** | | **Immediate word recall (*n*=1011)** | | **Delayed word recall (*n*=990)** | |
| --- | --- | --- | --- | --- | --- | --- |
|  | ***β* (95%CI)** | ***p*** | ***β* (95%CI)** | ***p*** | ***β* (95%CI)** | ***p*** |
| **Unadjusted model** |  |  |  |  |  |  |
| Self-esteem in 2014 | 0.09 (0.05,0.13) | <0.001 | 0.04 (0.02,0.06) | <0.001 | 0.04 (0.02,0.06) | 0.001 |
| **Adjusted model** |  |  |  |  |  |  |
| Self-esteem in 2014 | 0.06 (0.02,0.11) | 0.004 | 0.03 (0.01,0.05) | 0.007 | 0.03 (0.01,0.05) | 0.007 |

Note: In the adjusted model, this table controlled for adolescents’ characteristics (age and gender), parents’ characteristics (maternal age, paternal age, maternal education level, paternal education level, maternal employment status, paternal employment status), and household characteristics (urban‒rural location, household per capita income, family size).
